# Genetics and Epigenetics of Obsessive–Compulsive Disorder

**DOI:** 10.3390/genes17020189

**Published:** 2026-02-02

**Authors:** Federico Bernoni d’Aversa, Massimo Gennarelli

**Affiliations:** 1 Genetics Unit, IRCCS Istituto Centro San Giovanni di Dio Fatebenefratelli, 25125 Brescia, Italy; 2 Department of Molecular and Translational Medicine, University of Brescia, 25123 Brescia, Italy

**Keywords:** obsessive–compulsive disorder, genetics, epigenetics, endophenotypes

## Abstract

**Background**: Obsessive–compulsive disorder (OCD) is a heterogeneous psychiatric condition with substantial heritability. Early genetic studies were often underpowered and produced limited reproducibility, but recent large-scale genomic and multi-omic approaches are beginning to elucidate the genetic architecture of OCD. **Objectives**: This review aims to synthesise current evidence from recent genomic and epigenomic studies on OCD and their implications for molecular pathways of pathogenesis, including endophenotypes. **Methods**: We reviewed peer-reviewed literature and preprints published in recent years, focusing on multiple genetic approaches, including genome-wide association studies (GWAS), whole exome sequencing (WES), whole genome sequencing (WGS), and methylome-wide association studies (MWAS). We then integrated the results with endophenotypic evidence at the biochemical, physiological, structural, functional, and executive/cognitive levels. **Results**: Recent large-scale genomic studies provide strong evidence of a highly polygenic contribution from common variants, while rare coding and structural variants also contribute measurably, with enriched signals in pathways relevant to neurodevelopment and, in some cohorts, early-onset presentations. Epigenomic studies have moved from scattered findings to more replicable methylation patterns, including loci influenced by nearby genetic variation and indications of sex-dependent effects. Although convergence at the single-gene level remains limited, cross-study and cross-omics signals increasingly point to biological domains involving synaptic organisation and plasticity, neurological development and chromatin regulation, immune/stress pathways, and cellular homeostasis. **Conclusions**: The biology of OCD risk is best represented by an integrative model combining polygenic load, contributions from rare variants, and regulatory (epigenetic) mechanisms that influence intermediate phenotypes at the circuit and cognitive levels. The current findings are not yet clinically applicable for individual diagnosis; however, they may inform future multidisciplinary research frameworks and, in the longer term, contribute to the development of more personalised approaches in OCD.

## 1. Introduction

Obsessive–compulsive disorder (OCD) is a persistent and disabling psychiatric condition characterised by intrusive and unwanted thoughts (obsessions) and/or repetitive acts or rituals (compulsions) that cause significant distress and interfere significantly with daily life [1]. In addition to marked functional impairment, individuals with OCD are at increased risk of suicide, as demonstrated in large population cohorts [2].

With an estimated global lifetime prevalence of between 1 and 4% [3], OCD is one of the major public health problems among psychiatric conditions [4], with significant individual, economic, and social consequences.

OCD is widely considered a complex, multifactorial disorder resulting from the interaction between genetic vulnerability and environmental exposures.

Family, twin, and molecular genetic studies consistently indicate a substantial heritable component in OCD. First-degree relatives of affected individuals show a markedly increased lifetime risk, with an approximately fivefold increase reported in classical family studies [5] and relative risks around eightfold in large cohorts based on national registries [6]. Twin studies estimate heritability at approximately 40–50% [7], and a recent meta-analytic study confirms a substantial heritable component within a multifactorial framework that also involves environmental risk factors and gene-environment interactions, including miRNA [8]. In this context, epigenetic mechanisms such as DNA methylation and chromatin remodelling are increasingly recognised as plausible mediators linking environmental exposures to lasting changes in gene expression relevant to OCD [9].

Earlier genetic studies on OCD largely relied on candidate gene studies/approaches. These investigations have focused predominantly on polymorphisms in serotonergic, dopaminergic, and glutamatergic genes selected on the basis of pharmacological models and hypotheses about Cortico–Striato–Thalamic–Cortical (CSTC) circuits. Although some variants have shown nominal or meta-analytic associations with OCD [10,11,12,13], small sample sizes and often inconsistent results across cohorts make the single candidate gene approach insufficiently robust. Indeed, more recent Genome-Wide Association Studies (GWAS) have provided more consistent and replicable evidence, estimating that common single nucleotide polymorphisms (SNPs) account for only about 28–37% of the heritability [14]. In addition to common polygenic risk, there is growing evidence that other genetic causes contribute significantly to OCD susceptibility as rare functional variants, copy number variation, and indels.

The most studied classes of rare structural mutations are copy number variants (CNVs): several studies have reported an excess of rare and large CNVs in individuals with OCD, particularly when they affect genes that are highly intolerant to loss-of-function events [15]. Further studies on paediatric cohorts indicate that rare CNVs are particularly abundant in cases with early onset, suggesting a possible link with neurodevelopmental vulnerability [16]. In line with these findings, potentially deleterious CNVs (pdCNVs) have been reported to be present in approximately 9% of probands with OCD and are associated with specific symptom dimensions, particularly obsession and checking, supporting pdCNVs as part of the risk architecture and as modulators of the phenotypic expression of OCD [17]. Whole-Exome Sequencing studies have reported an excess of rare harmful variants, including deleterious missense SNVs/indels and loss of function, particularly in genes involved in synaptic organisation, chromatin structure, and neurological development [18,19].

These findings indicate that, although rare variants probably account for a smaller proportion of the heritability at the population level than common SNPs, they can exert large individual effects and are particularly relevant in OCD with neurological burden or early onset.

Complementarily, trio-based whole-exome and whole-genome sequencing studies have identified an excess of de novo deleterious and protein-truncating missense variants in probands with OCD [18,20,21]. Taken together, these findings outline a multilayered aetiological model in which common and rare variants—whether inherited or de novo and affecting coding or structural elements—contribute to OCD risk through distributed and interacting effects, helping to bridge the gap between polygenic predisposition and neurodevelopmental vulnerability.

Despite growing interest in objective biomarkers, the diagnosis of OCD is still based exclusively on the assessment of symptoms and observable behaviour [22]. Although solid neuroimaging evidence has consistently shown CSTC abnormalities at the group level, structural and functional imaging is not currently used as a diagnostic tool in clinical practice. Magnetic resonance imaging (MRI) studies of unaffected first-degree relatives further support the presence of heritable circuit-level differences [23], and large-scale meta-analyses demonstrate subcortical volumetric abnormalities in both paediatric and adult OCD [24]. To bridge the gap between genetic variation and clinically observable symptoms, research has increasingly focused on endophenotypes, intermediate biological signatures that lie between the genome and the phenotype. These include neurochemical profiles, CSTC circuit dynamics, structural neuroanatomical markers, executive control traits, and error monitoring processes.

Genetic imaging studies show that variation in glutamatergic genes influences the volume of the putamen and nucleus accumbens [25], while other studies link genetic variation, such as *COMT* and *BDNF* to alterations in decision-making, cognitive control, and error processing [26,27,28].

Taken together, these advances suggest that integrating common variant polygenic load, rare coding and structural mutations, neurocognitive endophenotypes, and circuit-level alterations may help refine a biologically coherent model of OCD.

The overall aim of this review is to provide an up-to-date, integrated overview of current knowledge on the genetics of obsessive–compulsive disorder (OCD), with a focus on recent and methodologically robust studies. We synthesise evidence on common small-effect variants, rare larger-effect variants associated with OCD, and emerging epigenomic findings. We also include endophenotypes to provide a framework for linking genetic and epigenetic results to multilevel intermediate phenotypes—biochemical, physiological, structural, functional, and cognitive/executive—thereby highlighting measurable markers that may help link genetic liability and clinical expression. Finally, we examine cross-study convergence across genomic, epigenomic, and endophenotypic approaches to identify overlapping signals and prioritise OCD-relevant genes and pathways.

## 2. Genomics

### 2.1. Genome Wide Association Studies (GWAS)

In 2013, Stewart et al. [29] published the first GWAS of clinically diagnosed OCD, which did not identify any genome-wide significant loci in the case–control analysis; only one trio-based signal near *BTBD3* reached genome-wide significance, but it was not replicated in larger meta-analyses nor did it become an established risk locus. Subsequent case–control GWAS of OCD up to 2020 failed to detect robust genome-wide significant variants, a pattern highlighted by reviews by Saraiva et al. [30] and Mahjani et al. [31], who attributed the negative results largely to limited sample sizes. In parallel, GWAS of obsessive-compulsive symptoms (OCS) in population cohorts reported significant genome-wide associations for *MEF2BNB* [32], but this locus has not been consistently replicated in clinical samples of OCD. More recently, Burton et al. [33] identified the first genome-wide significant variant for OC traits in *PTPRD* and found an association with OCD case–control status in a meta-analysis, providing a bridge between symptom-based phenotypes and clinical phenotypes.

Only with much larger case–control meta-analyses, culminating in the large meta-analysis by Strom et al. in 2025 [14], robust genome-wide significant loci for OCD diagnosis began to emerge, shifting the field from isolated suggestive signals to reproducible common variant risk loci. This case–control study, which included 53,660 cases and 2,044,417 controls, remains the largest GWAS on OCD to date, and identified 30 independent significant loci at the genomic level. Gene-based approaches highlighted 249 potential effector genes, from which 25 were extensively prioritised using multiple gene-mapping and omics-integration approaches. These candidates were further supported by colocalization analyses and heterogeneity testing within summary-data eQTL (expression quantitative trait loci) Mendelian-randomization frameworks (with *DALRD3* and *WDR6* supported by both methods, and *CTNND1* showing converging evidence in several gene-based and colocalisation-based tests). The strongest association was observed in a locus containing SNP rs78587207, where genetic analyses identified four potential causal genes: *CLP1* (11q12.1), *TMX2* (11q12.1), *ZDHHC5* (11q12.1), and *CTNND1* (11q12.1).

Many of the recently associated gene clusters in biological processes relevant to the pathophysiology of OCD, including synaptic and membrane signalling (e.g., *FLOT1*, *ZDHHC5*, *CTNND1*, *YWHAB*) and neuronal growth and synaptic development (e.g., *AURKB*, *MEF2C*, *LAMB2*), further support the central role of synaptic mechanisms and neurological development in the disorder. In addition, four priority genes mapped to the MHC locus (*TRIM27*, *TUBB*, *FLOT1*, and *IER3*) have been associated with OCD, further supporting the contribution of immunological mechanisms to the disorder. Notably, most of the significant genes identified in the GWAS by Strom et al. had not been implicated in previous genetic studies of OCD, highlighting how larger sample sizes and unbiased genome-wide approaches can reveal new risk pathways.

A summary of the priority genes in Strom’s work [14] and their main presumed biological domains is provided in Table 1.

### 2.2. Copy Number Variant (CNV) Studies

The first genome-wide CNV study that included OCD was published by McGrath et al. [34] in 2014, who analysed large and rare CNVs in a cohort of patients with various disorders, with confirmed diagnoses of OCD and Tourette syndrome. Although this work reported that OCD cases may have CNVs in neurodevelopmental regions, it did not detect a significant overall CNV burden nor identify any robust risk loci specific to OCD.

A broader, multidisciplinary view was provided by Zarrei et al. [35], who compiled an extensive resource of CNVs in various neurodevelopmental disorders (including autism spectrum disorder, attention-deficit/hyperactivity disorder, schizophrenia, and OCD) genotyped on the same high-resolution microarray platform. Clinically relevant CNVs were observed in approximately 10% of all cases and in approximately 5–6% of OCD cases, typically involving recurrent genomic regions already implicated in other neurodevelopmental syndromes. These data support a pleiotropic model in which OCD shares part of the CNV risk architecture with other neurodevelopmental disorders, rather than being characterised by unique CNV hotspots.

In line with this, the review by Saraiva et al. [30] concluded that, as of 2020, no robust, OCD-specific CNV loci had been identified, and that existing findings were based on small, heterogeneous samples without clear replication.

More recent and larger studies have begun to clarify the contribution of rare CNVs to OCD.

In a Swedish population-based cohort, Mahjani et al. [17] used chromosomal microarrays to identify potentially harmful CNVs in OCD and chronic tic disorders (CTDs), reporting pdCNVs in approximately 9% of probands with OCD and highlighting several neurodevelopmental loci, including recurrent events at 1q21.1, 16p13.3, and especially 16p13.11, where genes such as *NDE1* and *MIR484* are strong candidates.

A large Nordic case–control study by Halvorsen et al. [15] using SNP-array data showed an increase in the burden of rare and large CNVs in adult OCD cases compared to controls, although the specific loci only partially overlapped with those reported in previous studies.

Abdallah et al. [16] extended this work to paediatric pervasive developmental disorders (PDD; now largely encompassed within ASD) using CNV calls from whole exome sequencing (WES) trios, identifying 49 rare CNVs (21 deletions, 28 duplications) and 12 de novo CNVs (9 deletions, 3 duplications) overlapping 25 genes.

They reported a significant enrichment of rare de novo CNVs in affected children and prioritised genes such as *SMAD2*, *MDM2*, and *ANAPC1*, which are involved in cell proliferation and neurodevelopmental signalling. These findings suggest that rare de novo CNVs may be particularly relevant to early-onset and clinically severe forms of the disorder.

The genes showing the strongest and most biologically interpretable support in CNV studies are summarised in Table 2.

### 2.3. Rare Variants from Whole Exome and Whole Genome Sequencing (WES, WGS)

In addition to CNVs, several studies have investigated the contribution of rare coding single nucleotide variants (SNVs) and short insertions/deletions (indels) to OCD using Whole Exome and Whole Genome Sequencing.

These variants can be inherited from an unaffected parent or arise de novo in the proband, and most analyses have focused on rare deleterious mutations, including variants likely to alter gene expression (nonsense, frameshift, and essential splicing site) and missense alterations predicted to severely impair protein function.

Halvorsen et al. [19] performed WES in the largest OCD cohort to date, combining 1263 cases and 11,580 controls with 587 trios. In the case–control component, they evaluated rare harmful inherited coding variants and reported an increased burden of rare loss-of-function and predicted harmful missense variants in OCD, particularly in genes that are intolerant to loss-of-function mutations. This pattern is consistent with a highly polygenic rare variant architecture, in which many different genes each carry a small to moderate increase in risk rather than a few genes with large effects. In gene-based testing, the strongest single-gene association was observed for *SLITRK5*, a member of a family of genes that regulates the development of excitatory and inhibitory synapses within cortico-striatal circuits. Although the *SLITRK5* signal did not reach significance at the exome level after rigorous multiple testing correction and still requires replication, its suggested involvement is consistent with converging evidence of synaptic circuit and CSTC dysfunction in OCD [36,37].

Regarding de novo mutations, Cappi et al. [20], in 2016, performed a Whole-Exome Sequencing (WES) study on 20 OCD trios. Although the sample size was small, they reported an increased rate of de novo SNVs in probands compared to published de novo SNV rates in unaffected sibling controls. No single gene reached significance at the whole-exome level, but this study provided the first evidence that rare de novo coding variants may contribute to the risk of OCD.

In a larger trio WES study, Cappi et al. [18] found, in 2020, a significant enrichment of de novo missense variants that likely disrupt genes, and predicted damage in OCD probands compared to controls (184 OCD trios and 777 control trios). They identified two high-confidence candidate risk genes for OCD, *CHD8* and *SCUBE1*, each harbouring two independent deleterious de novo variants in unrelated probands. Based on their modelling, the authors estimated that harmful de novo variants in approximately 335 genes contribute to risk in approximately 22% of OCD cases, highlighting a substantial contribution of rare and highly penetrant de novo mutations to OCD, particularly in neurodevelopmental genes that overlap with autism spectrum disorder and related neurodevelopmental syndromes.

More recently, Guan Ning Lin et al. [21] applied whole-genome sequencing (WGS) to 53 trios of OCD to study rare de novo SNVs, indels, and structural variants across the entire genome. Using an evidence-based prioritisation framework, they highlighted several genes with strong aggregate support, including *SETD5*, *KDM3B*, and *ASXL3*, as well as a deleterious de novo structural variant that disrupts *FBL*. All four genes encode chromatin or epigenetic regulators, and harmful de novo mutations in these genes are known to cause severe neurodevelopmental syndromes with intellectual disability and autism spectrum features. Co-expression analyses further revealed altered patterns among these chromatin-modifying genes and their epigenetic regulators in the prefrontal cortex of individuals with OCD, suggesting that chromatin dysregulation may represent a convergent mechanism linking rare de novo mutations to the pathophysiology of OCD.

Taken together, these exome and genome sequencing studies suggest that rare coding variants, both inherited and de novo, contribute measurably to the risk of OCD.

A summary of the most supported genes in rare variant studies (WES/WGS) is provided in Table 3.

## 3. Epigenomics

### 3.1. Methylation

The first studies directly analysing DNA methylation in OCD patients are fairly recent, dating back to 2016, with an epigenomic association study (EWAS) on the Han population [38] and a candidate gene study based on bisulphite pyrosequencing [39], both of which failed to produce significant or replicable results at the genomic level.

Recent reviews have concluded that, until around 2020, epigenetic studies on OCD were small-scale and had not identified robust and replicable DNA methylation loci associated with the disorder [30,40].

In 2022, Schiele et al. [41] conducted the first epigenomic-wide association study (EWAS) of OCD in adults of European descent using whole blood. They identified nine new epigenomically significant quantitative trait methylation sites and 21 suggestive findings, with the main signals mapped to a region hosting *MIR12136* and mitochondrial pseudogenes. The effect sizes were modest and there was little overlap with the previous Chinese EWAS by Yue et al., but the study provided independent support for methylation changes distributed in neurobiologically relevant loci.

A major advance came from the two-stage EWAS performed on whole blood by Campos-Martín et al. [42]. Their discovery and replication design yielded 305 differentially methylated CpG sites that replicated across all cohorts, and a core set of 12 CpGs was used to derive a methylation profile score (MPS). The MPS discriminated OCD cases from controls, correlated with symptom severity on the Yale–Brown Obsessive Compulsive Scale (Y-BOCS), and showed preliminary associations with cognitive behavioural therapy (CBT) outcome, suggesting that methylation signatures may capture both disease predisposition and aspects of treatment response.

More recently, a large methylome-wide association study (MWAS) using salivary DNA provided the largest methylation dataset in OCD to date. Höffler et al. [43] identified 35 differentially methylated positions (DMPs) and 17 differentially methylated regions (DMRs) associated with OCD. These loci were mapped to genes involved in neurotransmission, neurological development, synaptic function, and immune regulation.

In particular, the strongest opposite-sex DMRs were annotated on *ARHGEF17*, *MUC2*, and *RIN1*, and sex-stratified analyses revealed additional DMRs with significant sex-methylation interactions at *TEX26*, *AKAP12*, *PIWIL1*, *PGBD5*, and *GABRB3*, suggesting a potentially sex-dimorphic epigenetic architecture. In this MWAS, analyses of methylation quantitative trait loci (mQTLs) showed that nearly half of the opposite-sex DMPs and more than half of the DMRs were influenced by nearby genetic variants and that at several loci, the same variants were associated with both methylation levels and OCD case–control status. This suggests that part of the methylation signal in this dataset represents a genetically anchored epigenetic correlate of OCD risk rather than a purely environmental imprint. At the same time, the cross-sectional design of saliva and the presence of loci without clear mQTLs imply that non-genetic exposures and disease-related processes also contribute to the observed methylation profiles, which are best interpreted as the combined result of genetic and environmental influences.

Genes annotated to significant DMPs/DMRs at the whole-methylome level in Höffler et al. (2025) [43] are grouped into major functional pathways in Table 4.

### 3.2. MicroRNAs

MicroRNAs (miRNAs) provide a mechanistically plausible layer through which genetic and environmental factors can influence the neural circuits and cognitive processes involved in obsessive–compulsive disorder (OCD). However, they currently do not meet classic endophenotypic criteria (heritability, familial aggregation, and trait-like stability), and all available data on OCD come from peripheral tissues with small sample sizes and limited replication. In this sense, miRNAs are best considered potential molecular modulators of neural and cognitive endophenotypes, rather than endophenotypes in their own right.

Experimental work on rodents has shown, for example, that brain-derived neurotrophic factor (BDNF) can enhance synaptic plasticity by upregulating miR-132, which promotes dendritic growth and spine formation in cortical neurons [44].

Yue et al. [45] reported increased plasma levels of miR-132 and miR-134 in adults with OCD compared to healthy controls.

Aydın et al. [46] subsequently analysed a panel of 12 candidate miRNAs and described a broader dysregulation pattern, with several miRNAs upregulated and others downregulated; in particular, increased expression of miR-106b-5p was reported to be associated with resistance to treatment with selective serotonin reuptake inhibitors (SSRIs).

More recent studies have partially replicated the dysregulation of miR-132 and introduced additional candidates: in female patients with OCD, Korkmaz et al. [47] observed an upregulation of miR-132 and a downregulation of miR-125b-5p, while Altunoz et al. [48] reported increased levels of miR-132-3p and correlations with symptom severity, linking this miRNA to both synaptic and immuno-inflammatory mechanisms.

In addition to case–control status, miRNAs may also modulate intermediate cognitive phenotypes. In a follow-up study, Aydın et al. [49] found that higher levels of miR-6740-5p were associated with better performance on the Tower of London task in patients with OCD, while in healthy controls, the same miRNA was negatively correlated with interference control in the Stroop test.

Although exploratory, these findings suggest that miRNAs may influence executive functions such as planning, cognitive flexibility, and interference control, domains that are consistently impaired in OCD.

Complementary transcription and neuroimaging work further supports the relevance of miRNA-regulated networks: Zhang et al. [50] identified sets of cortical genes whose expression patterns track OCD-related alterations in cortical thickness, many of which are known targets of miRNAs relevant to neurological development, including miR-132 and miR-134.

## 4. Endophenotypes

Endophenotypes are heritable and quantifiable traits that link genetic variation to clinical phenotypes through intermediate neurobiological levels. In OCD, six partially overlapping levels are informative: biochemical (molecular signalling; synaptic proteins), physiological (electrophysiology; circuit reactivity), structural (morphometry; microstructure), functional (system-level activation and connectivity), executive (inhibitory control, flexibility), and cognitive (learning and memory). Across these levels, dysregulation of the Cortico–Striato–Thalamic–Cortical (CSTC) circuit provides a unifying framework for interpretation [34,51,52]. An endophenotypic framework centred on genes and pathways aligns with current models linking genetic risk to CSTC network dysfunction in OCD [53].

In this review, we deliberately explore the endophenotypic landscape of OCD, rather than limiting ourselves to classical case–control genetics. To date, OCD-specific endophenotypic evidence remains relatively scarce and unevenly distributed across levels and genes, with substantial heterogeneity. To provide a mechanistic bridge between genetic variation and CSTC dysfunction, we have therefore adopted a graded evidence framework that integrates (i) OCD-specific human findings; (ii) human, non-OCD, or indirect/pathway-level evidence (e.g., imaging-genetics, cross-disorder, or pharmacogenetic signals that converge on biology relevant to CSTC); and (iii) preclinical data from animal and iPSC/cellular models when they offer unique mechanistic resolution. An overview of the experimental and clinical techniques used to assess endophenotypes at all levels is provided in Table 5.

**Figure 1:**
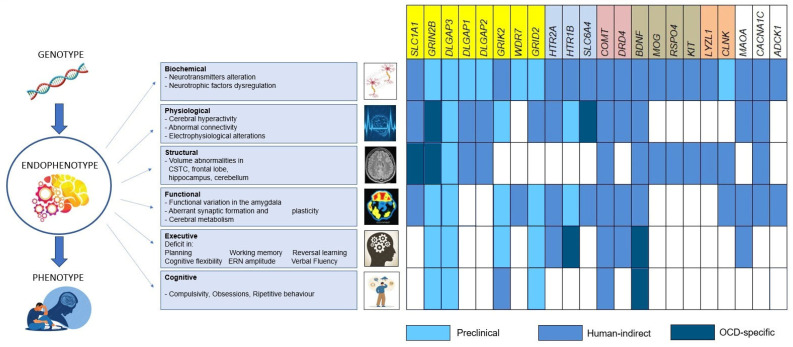
Between genotype and phenotype lies an intermediate level of biological organisation, commonly referred to as endophenotypes. Several genes identified in recent studies have been reported to be associated with specific psychiatric endophenotypes (often cross-disorder). Glutamatergic genes are highlighted in yellow; serotonergic genes in light blue; dopaminergic genes in violet; neurotrophic and neurodevelopmental genes in grey; immune-linked genes in pink; and other genes in white.

For clarity, the conceptual relationship between genotype, endophenotype, and clinical phenotype used in this chapter is illustrated in Figure 1.

Gene selection followed an integrative prioritisation approach. We started from genes and loci emerging as priorities in the large GWAS and gene-based approaches, from recurrent signals across rare-variant/CNV sequencing studies (Section 2.1, Section 2.2 and Section 2.3; Table 1 and Table 2) [14,15,16,17,18,19], and from loci highlighted by recent epigenomic and miRNA work when biologically coherent with CSTC circuit models (Section 3; Table 4) [40,41]. We also retained a small set of canonical neurotransmission and neuroplasticity candidates repeatedly discussed in OCD genetics literature and meta-analyses [10,11,12,13]. Genes were included in the present chapter when at least one endophenotypic level could be reasonably mapped using OCD-specific, indirect human, or preclinical evidence. Finally, genes were grouped into five clusters—glutamatergic, serotonergic, dopaminergic/monoaminergic, immune–neurodevelopmental, and neurodevelopmental—based on predominant biological function to facilitate mechanistic interpretation.

This integrative approach is designed to generate hypotheses while remaining biologically disciplined, making explicit where current knowledge is solid, where it is indirect, and where it is inferred from model systems, so that future work can fill the most significant information gaps. Where OCD-specific human evidence is limited, proposed gene–endophenotype links are presented as hypothesis-generating models informed by preclinical and indirect human data.

### 4.1. Glutamatergic Genes

Glutamatergic signalling and synaptic scaffolding are core components of Cortico–Striato–Thalamo–Cortical (CSTC) loop function, which is widely implicated in compulsive symptoms and cognitive rigidity in OCD. Accordingly, this section summarises key glutamatergic/synaptic candidates and their links to OCD-relevant endophenotypes across evidence levels.

***SLC1A1* (EAAT3).** *SLC1A1* encodes the neuronal glutamate transporter essential for synaptic reuptake within CSTC loops. Biochemically, *SLC1A1* variation is linked to glutamate transport dysfunction (supportive/indirect in humans) [54,55], with preclinical convergence on basal ganglia hyperactivity and stereotypy [56]. Physiologically, this may translate to increased cortico-striatal excitatory drive (supportive/indirect in humans) [55,56], while structurally, imaging-genetics work indicates thalamic volumetric alterations (Paediatric) [57]. Functionally, the model aligns with CSTC hyperactivity synthesised in imaging and MRS reviews (supportive/indirect in humans) [52]. Effects on executive and cognitive levels are inconsistent.

***GRIN2B* (GluN2B).** *GRIN2B* encodes an NMDA receptor subunit critical for Ca^2+^ permeability and plasticity. Variants affect channel properties and synaptic modulation (biochemistry, preclinical) [58], with physiological signatures of NMDA-dependent plasticity and regional shifts in glutamatergic concentration (anterior cingulate cortex (ACC)/orbitofrontal cortex (OFC)) [58]. Paediatric genetics and imaging reports link *GRIN2B* to ACC/OFC/thalamic volumes [58], converging on functional ACC hyperconnectivity in animal models [59]. Translational models indicate executive and cognitive deficits with reversal/set-shifting deficits (preclinical) [60].

***DLGAP3* (SAPAP3).** *DLGAP3* encodes a postsynaptic density (PSD) scaffold that connects NMDA-SHANK complexes within cortico-striatal circuits. Most evidence for *DLGAP3* comes from *Sapap3*−/− mouse models [61,62,63]. Biochemically, the loss of *SAPAP3* disrupts the NMDA–SHANK–SAPAP complex, impairing postsynaptic glutamatergic signalling and scaffold stability [61,64]. Physiologically, cortico-striatal long-term depression (LTD) is reduced, with increased excitatory drive and CSTC imbalance [61,64]. At the synaptic microstructural level, PSD disorganisation and loss of dendritic spines have been reported [62,64]. In *Sapap3−/−* mice, *SAPAP3* deficiency produces circuit-selective cortico-striatal synaptic insufficiency with OFC-striatal disconnection/hyperactivation, leading to compulsive grooming and impaired action selection/inhibitory (functional) control [61,63]. At the executive and cognitive level, mouse phenotypes indicate behavioural rigidity and impaired associative/extinction learning as proxy (preclinical) endophenotypes [62,63]. Evidence in human OCD remains limited [65,66].

***DLGAP1* (*SAPAP1*).** *DLGAP1* encodes a postsynaptic density scaffold that anchors NMDA-SHANK complexes, thereby organising glutamatergic signalling in cortico-striatal synapses. This biochemical scaffold is supported by preclinical synaptic biology and is consistent with a plausible physiological model in which NMDA–SHANK–SAPAP complex dysfunction may contribute to CSTC signal integration deficits (indirect in humans) [67]. Imaging-genetic links with ACC and thalamic volumes have been reported in large-scale datasets (indirect in humans) [68]. No replicated functional, executive, or cognitive effects specific to OCD are available.

***DLGAP2* (*SAPAP2*).** *DLGAP2* is a postsynaptic density scaffold homologous to *DLGAP1/3*, which organises NMDA-SHANK macromolecular complexes in glutamatergic synapses. At the biochemical level, preclinical evidence indicates a PSD scaffolding role, suggesting a plausible effect on glutamatergic signal integration (hypothesis-generating) [12]. Physiological endophenotypes have not yet been established. At the structural level, indirect signals have been reported in humans—within glutamatergic imaging/*SAPAP* family genetic frameworks—suggesting reductions in orbitofrontal/striatal white matter and a plausible CSTC link, although to date, there has been no specific replication for OCD (indirect in humans, at the pathway level) [25,68]. Functional, executive, and cognitive endophenotypes have not been established for *DLGAP2* in OCD.

***GRIK2* (GluK2; kainate receptor).** *GRIK2* modulates presynaptic release and synaptic plasticity. Biochemically, kainate receptor variation influences Glu/GABA modulation and plasticity (human support, indirect) [69,70]. Physiologically, evidence from GluK2 models indicates that kainate receptor perturbations alter circuit electrophysiology, modifying excitatory–inhibitory balance and short-term plasticity in hippocampal–cortical pathways and modulating NMDA-dependent responses (preclinical) [71].

Specific structural markers of OCD have not been consistently replicated. At the functional level, genetic meta-analytic frameworks suggest an alteration in CSTC synaptic/network gating efficiency (preclinical/pathway-level) [72], with executive signals in inhibition/flexibility also supportive [71] and supported by preclinical studies on the kainate receptor. Cognitively, associative learning and flexibility, including reversal learning, may be impaired (human, indirect support) [73].

***WDR7* (Rabconnectin-3 complex).** *WDR7* contributes to V-ATPase assembly and vesicular acidification, regulating neurotransmitter load. Biochemically, *WDR7* participates in vesicle acidification via V-ATPase regulation, supported by preclinical evidence from rat brain synaptic vesicles, zebrafish hair cells, and human/mouse cell systems [74,75,76,77]. Although OCD-specific physiological or structural signals are not replicated, a functional imbalance of the CSTC mediated by the presynaptic vesicle cycle has been inferred from genetic/pathway analyses (human, indirect) [25]. Effects on executive and cognitive levels are inconsistent.

***GRID2* (GluRδ2).** *GRID2*, expressed in Purkinje cells (PC) with functional projections to the prefrontal cortex (PFC), supports plasticity and timing. Biochemically, δ2 receptor signalling shapes synaptic organisation and parallel fibre–Purkinje cell long-term depression (PF-PC LTD) (preclinical) [78]. Physiologically, GluD2 disruption abolishes PF-PC LTD and disrupts cerebellar electrophysiology, shifting excitation/inhibition calibration and temporal coding in animal models [79]. Structural replication in OCD is lacking. Functionally, links to motor/compulsive control networks are supported at the pathway level and supported by preclinical δ2 data [80,81]. Executive control/inhibition in OCD involves fronto-cerebellar loops; the contribution of *GRID2* remains at the pathway level rather than being gene-centric [80]. Cognitively, repetitive/procedural rigidity remains at the preclinical/pathway level, with supporting (indirect) human genetic association from meta-analytic frameworks [81,82].

Together, these genes point to altered excitatory circuit tuning, which is further shaped by neuromodulatory systems—most notably serotonin—addressed next.

### 4.2. Serotonergic Genes

Serotonergic pathways are central to OCD treatment and modulate cortical excitability, affective reactivity, and CSTC gating. We highlight serotonergic candidates and the intermediate phenotypes most consistently implicated by human and preclinical evidence.

***HTR2A* (*5-HT2A*).** *HTR2A* encodes a postsynaptic 5-HT receptor that modulates cortical excitability and synaptic density. Biochemically, dysfunction of *5-HT2A* signalling may increase cortical excitability and remodels synaptic organisation (human, indirect) [83,84,85]. The most consistent physiological endophenotype is CSTC hyperactivation (human-indirect) [34,52,86], with functional systems support reporting OFC/vm PFC-striatal hyperactivation in OCD [34,52,86]. No replicated structural effects have been established. At the executive level, *5-HT2A* contributes to inhibition/affect interaction [87], while cognitive effects are not replicated.

***HTR1B* (*5-HT1B*).** *HTR1B* encodes a presynaptic autoreceptor that modulates 5-HT release and impulsivity. Biochemically, rs6296 and related variations influence receptor function and synaptic density (indirect in humans) [86,88]. Physiological and functional evidence, strongest in animal models, indicates OFC/striatal hyperactivation and altered fronto-striatal connectivity [89]. Structural and cognitive endophenotypes remain unreplicated; executive effects appear to be linked to impulsivity/aggressiveness, but must be considered context-dependent and indirect in humans [86]. Cognitive effects are not replicated.

***SLC6A4* (SERT).** *SLC6A4* encodes the serotonin transporter; promoter polymorphisms (5-HTTLPR) modulate transporter expression and influence serotonergic reuptake (biochemical) [90]. Altered SERT availability, particularly in thalamic/limbic nodes, is consistent with a serotonergic physiological signal endophenotype in OCD linked to *SLC6A4* (supportive/indirect) [91,92]. Functionally, altered OFC/ACC-striatal responses during inhibition/feedback paradigms have been described in OCD, but direct 5-HTTLPR × fMRI interactions are inconsistent; therefore, we consider this evidence supportive/indirect [93,94,95,96]. No replicated structural or cognitive endophenotypes for *SLC6A4* in OCD have been established.

Because serotonergic modulation interacts with reinforcement and action control, the next section focuses on dopaminergic/monoaminergic contributions.

### 4.3. Dopaminergic Genes

Dopaminergic signalling shapes action selection, habit learning, and error monitoring—processes repeatedly linked to compulsivity. Below we summarise candidate genes and their putative endophenotypic associations, while acknowledging the heterogeneity of OCD-specific human findings.

***COMT* (Val158Met).** *COMT* encodes catechol-O-methyltransferase and controls prefrontal dopaminergic tone. Biochemically, the Met allele reduces enzyme activity by approximately 40%, increasing prefrontal DA [12,97]. Physiologically, genotype and pharmacological challenges modulate Error-Related Negativity and Feedback-Related Negativity (ERN/FRN) [98,99]. Structural associations (hippocampus, dorsolateral prefrontal cortex (DLPFC), default mode network) are largely cross-sectional [26,100]. Functionally, differences in fronto-striatal control are inferred from physiology; executive variation in working memory and stress-sensitive executive performance has been reported [97], with no robust OCD-specific cognitive effects [97,101,102].

***DRD4 (D4).*** *DRD4* encodes the dopamine D4 receptor, with polymorphisms that alter PFC/striatal signalling. Biochemically, the variation in D4 signalling is well characterised [103]. Physiologically, *DRD4* modulates error monitoring (ERN/FRN) [104]. No replicated structural effects have been established; functionally, the gene is related to differences in action regulation and altered CSTC dynamics [104]. Executive effects include impulsivity traits [104], with no replicated cognitive associations.

Overall, we propose that dopaminergic candidates may act as modulators of CSTC gain and the prioritisation of internal and external signals, helping to contextualise OCD-relevant intermediate phenotypes even when gene-level associations remain modest and inconsistently replicated.

### 4.4. Neurotrophic and Neurodevelopmental Genes

Neurodevelopmental and glial-regulatory processes may shape synaptic remodelling and network homeostasis, with potential consequences for CSTC circuit vulnerability. This section summarises representative candidates in this broader regulatory domain and maps their evidence to OCD-relevant endophenotypes.

***BDNF* (Val66Met).** *BDNF* encodes an essential neurotrophin for synaptic plasticity; Val66Met reduces activity-dependent (biochemical) secretion [105]. Physiologically, error processing can be modulated by gene-environment interactions [28]. Structural differences in hippocampal and cortical measurements span multiple domains [105]; functionally, differences in CSTC connectivity are consistent with neuroplastic mechanisms [105]. Executive (and memory) variability has been documented in cohorts of patients with OCD [106], projecting onto cognitive alterations in verbal memory and planning [105].

***MOG*.** *MOG* encodes an oligodendrocyte myelin glycoprotein. Its biochemical role is in myelin biology (indirect in humans) [107]; physiological effects are indirect (conduction). Structurally, an association with increased total white matter volume in OCD has been reported, and large-scale genetic imaging analyses suggest concordance between genetic risk for OCD and subcortical volumes [25,107]. Although *MOG* is genetically associated with OCD [107], a replicated, *MOG*-focused imaging genetic link is not yet available; therefore, we classify *MOG*-structural as indirect in humans [24,50,107] without replicated functional, executive, or cognitive signals.

***RSPO4*.** *RSPO4* is an agonist of Wnt/β-catenin (via LGR4/5/6; inhibition of ZNRF3/RNF43). Biochemically, it enhances Wnt signalling [108,109]; physiologically, its effects are evolutionary/indirect in nature. Structurally, imaging genetic variants near *RSPO4* and putamen volume are jointly associated with OCD risk [25]. Functional, executive, and cognitive effects remain unreplicated.

***KIT*** (c-*KIT*; tyrosine kinase receptor). *KIT* encodes a tyrosine kinase receptor activated by stem cell factor (SCF) that governs neurodevelopmental processes (neuronal proliferation/migration, axon guidance, synaptic maturation). At the biochemical level, *KIT* variation shapes an endophenotype of impaired neurodevelopmental receptor tyrosine kinase (RTK) signalling [13]. OCD-specific physiological markers have not yet been replicated. Structurally, large-scale genetic imaging suggests—at the pathway level—thalamic and prefrontal changes consistent with evolutionary remodelling of CSTC architecture [25]. Overall, the relevance of KIT should be considered emerging.

Taken together, these candidates suggest that upstream regulatory mechanisms may influence OCD-relevant circuitry indirectly, reinforcing the value of endophenotype-level interpretation when diagnosis-level associations are heterogeneous.

### 4.5. Immune-Linked Genes

Immune-related pathways have been increasingly discussed in OCD as potential modulators of brain function and symptom expression, although the specificity of the findings remains variable. Here, we summarise immune-linked candidates reported in genetic and imaging-genetic studies and map them to OCD-relevant endophenotypes.

***LYZL1*.** *LYZL1* encodes a lysozyme-like protein involved in innate immunity. Biochemically, it exerts bacteriolytic functions [13]; its physiological effects are indirect through neuroimmune crosstalk. Structurally, an upstream variant has been associated with amygdala volume and OCD risk in imaging genetic analyses [25]. Currently, there is a lack of functional, executive, and cognitive evidence.

***CLNK*.** *CLNK* encodes a cytokine-dependent adaptor in immune cell tyrosine kinase cascades. Biochemically, it integrates immune receptor signalling [110]; its physiological effects on the CNS are indirect, within a CSTC neuroinflammatory framework. Functionally, although there are no CLNK × fMRI readouts, convergent evidence in humans of low-grade immune dysregulation in OCD suggests that a cytokine-dependent adaptor like *CLNK* could modulate CSTC excitability and network allocation at the pathway level (indirect in humans) [13,20]. Structurally, genetic imaging links *CLNK* variation to thalamic volume [25]. Other endophenotype levels have not been replicated.

Taken together, these immune-linked candidates provide hypothesis-generating support for indirect modulation of OCD-relevant intermediate phenotypes, but their diagnostic specificity and causal role remain to be established.

### 4.6. Other Genes

Several reported candidates do not fit the main functional clusters or show heterogeneous evidence. Here, we briefly list these additional genes and map their putative endophenotypic links using the same graded framework.

***MAOA*.** *MAOA* encodes a monoamine oxidase that degrades 5-HT/DA/NE. The biochemical endophenotype focuses on monoaminergic turnover [111,112]; physiologically, the *MAOA* genotype modulates stress reactivity in the amygdala/ACC outside OCD [113]. In OCD-relevant contexts, *MAOA* may influence affective regulation and, indirectly, cognitive control/error monitoring through the prefrontal-cingulate circuit (functional) [114,115], with possible links to impulsivity/aggression (executive) [114,116]; however, this inference is based on cross-disorder imaging genetic evidence rather than specific replication in OCD, so we maintain a qualitative mention. Structural and cognitive evidence is insufficient.

***CACNA1C* (Cav1.2).** *CACNA1C* encodes an L-type Ca^2+^ channel that shapes cortico-limbic gating. Biochemically, it governs Ca^2+^ influx and downstream signalling [117]. Physiologically, variation impacts affective fear reactivity and prefrontal-amygdala gating [117]. Circuit-level structural and functional differences are cross-disorder but biologically coherent and hypothesis-generating to OCD, given the Ca^2+^-dependent regulation of CSTC/limbic systems [117]. Executive and cognitive endophenotypes are not included in the table due to a lack of specific OCD replication.

***ADCK1*** (COQ8A-like). *ADCK1* belongs to a family of mitochondrial kinases involved in coenzyme Q biosynthesis and bioenergetics. Therefore, biochemical variation in this gene is hypothesised to influence oxidative phosphorylation/ATP supply and synaptic energy homeostasis (indirect in humans) [118,119]. Functionally, the gene appears at a discovery level in genetic/meta-analytic studies with plausible effects on network-energy coupling [82], without established executive or cognitive endophenotypes.

Collectively, these additional candidates broaden the endophenotypic landscape of OCD, but the available evidence remains heterogeneous and largely hypothesis-generating.

## 5. Discussion

A key question addressed in this review is whether signals from GWAS/rare-variant studies, methylome and epigenome-wide association studies (MWAS/EWAS), and endophenotype-based mapping converge on shared genes or pathways.

At present, strict gene-level convergence across these approaches appears limited, which is not unexpected given the highly polygenic architecture of OCD, differences in statistical power, and the tissue- and state-dependence of methylation signals. Nevertheless, cross-omic signals do emerge in OCD datasets (e.g., *AKAP12* and *GABBR1* reported across sequencing- and methylation-based approaches), suggesting that a subset of candidates may be captured by multiple layers of evidence. Importantly, even when gene-by-gene overlap is modest, convergence becomes clearer at the pathway level—particularly for synaptic regulation and CSTC-relevant circuit mechanisms—supporting the value of integrative, multi-omics approaches combined with deep phenotyping. Ancestry composition and cohort characteristics across studies are summarised in Table 6.

A recent large cross-disorder analysis of common-variant liability across 14 psychiatric disorders provides an important framework for interpreting the limited single-gene convergence observed in OCD [120]. In that study, genome-wide overlap was parsimoniously captured by five genomic factors and hundreds of pleiotropic loci, indicating that a substantial fraction of genetic risk is shared across diagnostically distinct conditions. Notably, OCD loaded on a ‘Compulsive disorders’ factor that was driven primarily by shared liability to anorexia nervosa and OCD (with weaker contributions from Tourette syndrome and anxiety). This supports the view that part of the OCD common-variant signal reflects broader compulsivity-related dimensions rather than disorder-specific effects. Consistent with our synthesis, convergence emerged more clearly at the level of biological systems than at individual genes, with the transdiagnostic signal enriched for broad regulatory processes and factor-level differences mapping onto neurobiologically interpretable cellular signatures. These findings complement OCD-focused GWAS and multi-omics results by contextualising heterogeneity and supporting endophenotype-based stratification, while also highlighting current limitations related to ancestry representation and the restricted scope of common-variant analyses.

Because most OCD GWAS and sequencing cohorts remain enriched for European ancestry, effect sizes and risk loci may not transfer reliably to other populations, which can reduce polygenic risk score accuracy and bias locus discovery, and complicate rare-variant interpretation due to differences in allele frequencies and linkage disequilibrium across ancestries.

Within this cross-disorder context, we next highlight gene-level candidates that appear most informative in OCD, prioritising recurrence across studies, cross-omics support, and coherence with CSTC-relevant endophenotypes.

Several genes considered priorities by Strom et al. [14], although largely not highlighted as robust OCD candidates in previous association studies, have well-established roles in severe neurodevelopmental disorders (cross-disorder evidence), providing independent functional annotation consistent with convergence on neurodevelopmental, synaptic, and immune pathways. *MEF2C* haploinsufficiency causes a characteristic neurodevelopmental syndrome with global developmental delay/intellectual disability, seizures, stereotyped movements, and, in some cases, brain abnormalities [121,122]. Heterozygous pathogenic variants in *TUBB* have been reported in complex cortical malformation phenotypes (complex cortical dysplasia with other brain malformations), within the broader spectrum of tubulinopathies [123,124]. Mutations in *CLP1* cause pontocerebellar hypoplasia type 10, a severe early-onset neurodevelopmental disorder with progressive microcephaly, spasticity, seizures, and structural brain anomalies [125,126]. Recessive mutations in *LAMB2* cause Pierson syndrome, and experimental models support its role in neuromuscular and developmental processes [127,128,129]. In addition, *ARIH2* has a recognised role in immune regulation, including negative regulation of NLRP3 inflammasome activity, and rare human variants have been reported in neurodevelopmental phenotypes such as ASD/ID [130,131]. Although these syndromic associations cannot be directly extrapolated to OCD (given differences in variant class, penetrance, and phenotypic expressivity), they reinforce the idea that OCD risk biology converges on neurodevelopmental, synaptic, and immune pathways shared across neuropsychiatric conditions. Given the limited replication of individual genes typical of polygenic disorders, our aim is not to designate strictly “causal” genes, but rather to prioritise candidates most likely to link molecular variation to circuits and endophenotypes relevant to OCD; accordingly, we summarise below the genes that appear most informative for future mechanistic and stratified follow-up.

*DLGAP1* has emerged as a recurring candidate across multiple genetic studies of OCD [29,132,133,134], supporting its prioritisation despite limited genome-level convergence in previously underpowered datasets. In an endophenotypic context, the most defensible inference is a physiological/functional signature of the CSTC, conceptualised as dysfunction of the NMDA-SHANK-SAPAP complex leading to deficits in CSTC glutamatergic signal integration, with further indirect human support from genetic-imaging links with ACC and thalamic volumes. Notably, replicated functional, executive, or cognitive endophenotypes specific to OCD remain unavailable, and *DLGAP1* is not among the 25 top-priority genes in the latest large-scale GWAS, suggesting that its contribution may reflect locus heterogeneity and/or pathway-level effects rather than a consistently replicated monogenic signal.

*CHD8* stands out among genes frequently reported in OCD genetic studies because its strongest support comes from a rare de novo variant rather than association with a common variant. In the literature on rare variants summarised in this review, trio-based sequencing studies consistently implicate neurodevelopmental and chromatin regulation mechanisms, with Cappi et al. [18] identifying *CHD8* (alongside *SCUBE1*) as high-confidence candidate risk genes for OCD, each carrying two independent damaging de novo coding variants in unrelated probands. This finding is biologically consistent with broader evidence that de novo risk in OCD overlaps with ASD/NDD-related gene networks, and that chromatin dysregulation may represent a convergent pathway linking high-impact mutations to OCD vulnerability. From an endophenotypic perspective, *CHD8* is better framed at the pathway level: disruption of chromatin-mediated neurodevelopmental programmes would be expected to influence circuit maturation and network-level organisation, providing a plausible (indirect in humans) bridge to intermediate structural and functional phenotypes relevant to CSTC, although *CHD8*-centric endophenotypic readouts specific to OCD have yet to be established.

*AKAP12* and *GABBR1* represent notable examples of cross-omic convergence within this review, having been reported in a trio OCD whole-genome sequencing (WGS) study [21] and independently highlighted in a saliva methylome-wide association study (MWAS) [43]. In the MWAS, *GABBR1* maps to regions differentially methylated by sex, while *AKAP12* emerged in sex-stratified analyses (male-specific DMR), suggesting that part of the regulatory signal may be sex-dependent. Although peripheral methylation signatures cannot be directly extrapolated to brain tissue, the presence of genetically influenced methylation effects (mQTL-linked loci) supports the plausibility of genetically anchored regulatory correlates. From an endophenotypic perspective, *GABBR1* provides a direct mechanistic link to inhibitory neurotransmission and CSTC “gating” phenotypes (physiological/functional and executive control), while *AKAP12* is better framed as a candidate signalling scaffold potentially capable of influencing synaptic plasticity and network-level regulation, justifying integrative follow-up using neuroimaging and inhibitory control paradigms relevant to CSTC.

Recent CNV studies collectively suggest a modest increase in the burden of rare CNVs, often relevant to neurodevelopment, in OCD, with recurrent involvement of loci such as 16p13.11. It is important to emphasise that this signal should be interpreted at the locus level: CNVs spanning 16p13.11 typically impact multiple genes, and gene-level assignment remains uncertain. In this context, *MYH11*, located within 16p13.11, recurs in studies mainly because it falls within the CNV region; its established role in vascular smooth muscle function makes it a less intuitive CSTC/synaptic candidate, reinforcing the idea that 16p13.11 likely reflects multigenic dosage effects and a pleiotropic predisposition to neurodevelopment rather than a monogenic OCD mechanism.

*SCUBE1* is noteworthy because it represents a rare variant signal with relatively high specificity: in trio whole-exome OCD sequencing, it was highlighted along with *CHD8* as a high-confidence de novo candidate [18]. Experimental evidence links SCUBE1 domains to growth factor signalling (including BMP modulation) and neurodevelopmental morphogenesis [135,136]. Although OCD-specific endophenotypes have not yet been established for *SCUBE1*, its biology motivates testable intermediate phenotypes that span neurodevelopmental predisposition and neurovascular processes, potentially contributing to the stratification of OCD subgroups enriched for rare variants.

*BDNF* is best understood as an endophenotypic anchor focused on neuroplasticity in OCD (error monitoring, cognitive control/memory, and connectivity relevant to the CSTC), while gene-level association with OCD risk remains inconsistent, suggesting a modulatory rather than a primary role in risk.

Several genes recurring in previous OCD genetic literature also consistently map onto multilevel endophenotypes, reinforcing glutamatergic and synaptic plasticity as a central mechanistic axis. *DLGAP3* is the most mechanistically anchored example: its disruption is associated with deficits in synaptic plasticity and CSTC circuit dynamics, providing a biologically coherent bridge from the breakdown of the molecular scaffold (biochemical/physiological) to CSTC dysfunction (functional) and compulsivity-related control phenotypes (executive proxies), although gene-centric endophenotypic evidence specific to human OCD remains limited. Similarly, *SLC1A1* supports an endophenotypic profile for glutamate homeostasis: variation in glutamate transport is more readily linked to intermediate biochemical and physiological phenotypes (synaptic glutamate clearance and excitatory drive), indirect human imaging genetic signals in support, and a plausible functional interpretation within CSTC hyperactivity frameworks, while executive and cognitive outcomes remain inconsistent and should be considered secondary.

*GRIK2* further reinforces intermediate phenotypes related to plasticity. At all levels, it is best understood as a modulatory candidate influencing glutamatergic signalling and synaptic plasticity (biochemical/physiological), with pathway-level implications supporting CSTC gating and behavioural flexibility. In this context, intermediate cognitive phenotypes—particularly associative learning and flexibility—offer a plausible bridge toward rigidity and perseveration relevant to OCD, albeit with limited OCD-specific replication. Finally, *GRID2* extends the endophenotypic landscape beyond “canonical” CSTC nodes, pointing to cerebellar synaptic plasticity and network timing/control mechanisms. Although gene-centric evidence specific for OCD remains largely indirect, *GRID2* is useful at the pathway level for supporting models in which cerebellar contributions to cognitive control and habit-like learning interact with CSTC circuits, in line with intermediate phenotypes related to automatization, timing, and behavioural rigidity.

In exome case–control analyses, *SLITRK5* emerged as one of the strongest gene-level signals for the burden of rare damaging variants (although it did not reach exome-wide significance after correction). Given the role of the *SLITRK* family in synaptic adhesion and cortico-striatal circuit development, *SLITRK5* provides a biologically coherent and CSTC-relevant candidate, linking hereditary rare variants to intermediate phenotypes of action regulation and inhibitory control, justifying replication in larger sequencing cohorts and integration with circuit-level endophenotypes.

Overall, current results suggest that reported miRNA alterations in OCD are heterogeneous, with partial convergence on a small subset of candidates (miR-132/132-3p, miR-134, miR-125b-5p, miR-106b-5p, and miR-6740-5p) [45,46,47,48,49] previously linked in synaptic plasticity [44,45], monoaminergic signalling [47], immuno-inflammatory regulation [48], and executive functioning [49]. However, all available studies rely on relatively small samples, use targeted candidate panels rather than unbiased whole-miRNome profiling, focus on peripheral tissues, and show limited replication across cohorts, often with transdiagnostic rather than OCD-specific signals [30,38]. In this context, the miRNAs summarised in this review should be considered as putative molecular modulators of neural and cognitive endophenotypes rather than as established endophenotypes or biomarkers.

## 6. Conclusions

Over the past decade, OCD genetics has shifted from underpowered candidate-gene reports to large-scale genome-wide and multi-omics studies, which have only recently begun to yield statistically robust and biologically interpretable findings. Current evidence supports a highly polygenic architecture for common variation, alongside a measurable contribution of rare coding and structural variants—particularly in early-onset cases or presentations with higher neurodevelopmental burden. In parallel, epigenomic studies have moved from scattered signals to more replicable methylation patterns, including genetically anchored components (mQTL-linked) and emerging indications of sex-dependent effects.

At the single-gene level, convergence across studies remains limited, consistent with extensive locus heterogeneity; however, convergence is clearer at the level of biological systems. Across genomics and epigenomics, recurrent domains include synaptic organisation and plasticity within CSTC-relevant circuits, neurodevelopmental and chromatin-mediated regulation, immune/stress-related processes, and cellular homeostasis.

Looking forward, further progress will likely depend on harmonised phenotyping and standardised endophenotype batteries integrated with multi-omics. Integrative approaches combining genomic, transcriptomic, and epigenomic data may help identify convergent molecular signatures and generate testable hypotheses on how inherited variation relates to gene expression and epigenetic regulation in OCD-relevant circuits, thereby supporting mechanistic refinement and future research efforts aimed at biologically informed subgrouping.

Despite these advances, current genetic and epigenetic findings are not yet clinically actionable at the individual level; rather, their main value lies in refining mechanistic models and informing stratification into biologically informed OCD subgroups.

## Figures and Tables

**Table 1:** Prioritised genes from 2025 GWAS and their main biological functions. Collectively, the 30 loci converge on a limited set of biological domains, including synaptic and cell-adhesion signalling, neurodevelopmental and cytoskeletal organisation, mitochondrial and metabolic homeostasis, and ubiquitin–immune regulatory pathways. This pattern supports a model in which OCD risk arises from the combined disruption of synaptic integration, circuit development, cellular energy balance, and immune-related proteostatic control.

Gene	Name	Function	Pathway
*SLC25A17*	Solute carrier family 25 member 17	Peroxisomal transporter of CoA and related cofactors involved in lipid and oxidative metabolism.	Peroxisomal/mitochondrial metabolism
*ATP5MC1*	ATP synthase membrane subunit c locus 1	Subunit of mitochondrial ATP synthase proton channel required for oxidative phosphorylation and ATP production.	Mitochondrial oxidative phosphorylation
*ZDHHC5*	zDHHC palmitoyltransferase 5	Palmitoyltransferase that controls membrane localization and trafficking of synaptic and signalling proteins.	Palmitoylation/synaptic trafficking
*IER3*	Immediate early response 3	Stress-inducible regulator of cell survival and apoptosis in immune and inflammatory signalling.	Stress response/immune–apoptotic signalling
*CCDC71*	Coiled-coil domain containing 71	Coiled-coil scaffold protein implicated in cytoskeletal organisation and intracellular signalling complexes.	Cytoskeleton/scaffold
*XPNPEP3*	X-prolyl aminopeptidase 3	Mitochondrial metallopeptidase involved in peptide processing and ciliary/renal function.	Mitochondrial/ciliary function
*ACSF2*	Acyl-CoA synthetase family member 2	Acyl-CoA synthetase contributing to mitochondrial fatty-acid activation and lipid metabolism.	Lipid metabolism/mitochondria
*CTNND1*	Catenin delta 1	Catenin family adaptor linking cadherin-mediated cell adhesion to intracellular signalling and cytoskeletal dynamics.	Cell adhesion/junctional signalling
*MEF2C*	Myocyte enhancer factor 2C	Transcription factor regulating neuronal differentiation, synaptic plasticity, and broader neurodevelopmental programmes.	Transcriptional regulation/neurodevelopment
*KLHDC8B*	Kelch domain containing 8B	Kelch-repeat β-propeller protein organising protein complexes during mitosis and cell-cycle progression.	Cytoskeletal/cell-cycle regulation
*YWHAB*	Tyrosine 3-monooxygenase/tryptophan 5-monooxygenase activation protein beta	Phosphoserine-binding 14-3-3 adaptor that integrates kinase signalling and cell-cycle control.	Intracellular/synaptic signalling
*UBE2Z*	Ubiquitin conjugating enzyme E2 Z	Ubiquitin-conjugating enzyme (E2) that tags substrates for proteasomal degradation and signalling regulation.	Ubiquitin–proteasome/signalling
*TRIM27*	Tripartite motif containing 27	RING-type E3 ubiquitin ligase involved in transcriptional repression and developmental signalling pathways.	Transcriptional repression/ubiquitin signalling
*ARIH2*	Ariadne RBR E3 ubiquitin protein ligase 2	RBR E3 ubiquitin ligase implicated in Hedgehog signalling, immune regulation and protein quality control.	Ubiquitin ligase/immune–developmental
*DALRD3*	DALR anticodon binding domain containing 3	tRNA-binding protein thought to modulate translation and RNA metabolism.	RNA metabolism/translation
*PABPC1L*	Poly(A) binding protein cytoplasmic 1 like	Cytoplasmic poly(A)-binding protein controlling mRNA stability and translation during early development.	mRNA stability/translation
*CLP1*	Cleavage factor polyribonucleotide kinase subunit 1	RNA kinase in tRNA splicing and pre-mRNA 3′-end processing; essential for normal neurodevelopment.	RNA processing/neurodevelopment
*TUBB*	Tubulin beta class I	β-tubulin isoform forming microtubules critical for neuronal morphology and axonal transport.	Cytoskeleton/microtubules
*LAMB2*	Laminin subunit beta 2	Laminin β2 subunit in basement membranes, mediating cell adhesion, neurite outgrowth, and synapse stabilisation.	Extracellular matrix/synaptic connectivity
*WDR6*	WD repeat domain 6	WD-repeat scaffold protein interacting with LKB1 and implicated in growth and metabolic signalling.	Signalling scaffold/growth regulation
*AURKB*	Aurora kinase B	Serine/threonine kinase controlling chromosome segregation and mitotic spindle dynamics.	Cell-cycle/mitosis
*TMX2*	Thioredoxin related transmembrane protein 2	ER-resident thioredoxin-like protein involved in redox-dependent protein folding at mitochondria-associated membranes.	ER stress/redox homeostasis
*FLOT1*	Flotillin 1	Membrane-raft protein participating in endocytosis, vesicle trafficking, and organisation of signalling microdomains.	Membrane microdomains/vesicle trafficking
*P4HTM*	Prolyl 4-hydroxylase, transmembrane	ER prolyl-4-hydroxylase regulating HIFα stability and cellular responses to oxygen tension.	Hypoxia/HIF signalling
*MAIP1*	Matrix AAA peptidase interacting protein 1	Mitochondrial matrix protein supporting ribosome binding and calcium-dependent mitochondrial homeostasis.	Mitochondrial function/Ca^2+^ homeostasis

**Table 2:** Genes with the strongest and most biologically interpretable evidence across rare CNV studies in OCD.

Gene	Full Name	Function (Short)	Pathway	Study
*NDE1*	Nuclear distribution element 1	Centrosomal/microtubule-associated protein required for neuronal proliferation, migration and cortical development.	Neurodevelopment/microtubule–centrosome	Mahjani et al., 2022 [17]
*MIR484*	MicroRNA 484	Brain-expressed miRNA at 16p13.11 that modulates neurogenesis and protocadherin-19 signalling in experimental models.	miRNA regulation/neurodevelopment	Mahjani et al., 2022 [17]
*SMAD2*	SMAD family member 2	Intracellular effector of TGF-β signalling controlling cell proliferation, differentiation, and early neurodevelopment.	TGF-β/neurodevelopment	Abdallah et al., 2025 [16]
*MDM2*	MDM2 proto-oncogene, E3 ubiquitin ligase	Negative regulator of p53 that controls cell-cycle progression and apoptosis, influencing cortical proliferation/survival.	Cell-cycle/p53–apoptosis	Abdallah et al., 2025 [16]
*ANAPC1*	Anaphase-promoting complex subunit 1	Core component of the APC/C E3 ubiquitin ligase complex required for mitotic progression and neurodevelopmental timing.	Cell-cycle/ubiquitin ligase	Abdallah et al., 2025 [16]

**Table 3:** Genes with the strongest and most biologically interpretable evidence emerged across rare variant studies in OCD.

Gene	Full Name	Function (Short)	Pathway	Study
*CHD8*	Chromodomain helicase DNA-binding protein 8	Chromatin-remodelling factor that regulates large neurodevelopmental gene networks; high-confidence ASD/neurodevelopmental disorder (NDD) risk gene.	Chromatin remodelling/neurodevelopment	Cappi et al., 2020 [18]
*SCUBE1*	Signal peptide, CUB domain and EGF-like domain-containing protein 1	Secreted EGF-related glycoprotein involved in early CNS and vascular development and growth-factor signalling.	Growth-factor signalling/neurovascular	Cappi et al., 2020 [18]
*SLITRK5*	SLIT and NTRK-like family member 5	Postsynaptic adhesion molecule regulating excitatory/inhibitory synapse formation within cortico-striatal circuits.	Synaptic adhesion/CSTC signalling	Halvorsen et al., 2021 [19]
*SETD5*	SET domain-containing protein 5	Histone lysine methyltransferase that regulates broad neurodevelopmental transcriptional programmes; LoF causes NDD with ID/ASD.	Chromatin modification/neurodevelopment	Lin et al., 2022 [21]
*KDM3B*	Lysine demethylase 3B	H3K9 histone demethylase essential for epigenetic control of transcription; pathogenic variants cause Diets–Jongmans syndrome.	Epigenetic regulation/transcription	Lin et al., 2022 [21]
*ASXL3*	Additional sex combs-like protein 3	Scaffold for chromatin-remodelling complexes; truncating variants cause Bainbridge–Ropers syndrome with severe NDD.	Chromatin remodelilng/epigenetic control	Lin et al., 2022 [21]
*FBL*	Fibrillarin	Core nucleolar 2′-O-methyltransferase of box C/D snoRNPs linking rRNA modification, ribosome biogenesis and transcriptional control.	Ribosome biogenesis/RNA–chromatin interface	Lin et al., 2022 [21]

**Table 4:** Genes from Höffler et al. (2025) [43] are grouped into five broad biological domains based on their predominant function; some loci have pleiotropic roles across multiple pathways.

**Epigenetic/chromatin/transcriptional regulation** *DNMT3A*, *DAXX*, *GADD45A*, *CBFA2T3*, *FAM120B*, *HIVEP3*, *HEMK1*, *RBM47*, *LINC00511*, *LINC01271*, *LINC01996*, *RN7SL363P*, *RPL17P34*, *ZNF833P*, *MIR29A*, *MIR21*, *MIR4489*, *HNRNPA1P10*, *EEF1A1P49*
**Neurotransmission and CSTC synaptic signalling** *GABBR1*, *GABRB3*, *GPRIN3*, *RIN1*, *ADGRB1 (BAI1)*, *ARHGEF17*, *ARHGEF10*, *ZNRF1*, *SLC12A7 (KCC4)*, *KIFC3*
**Neurodevelopment, cell polarity and structural plasticity** *DCHS1*, *TUBGCP3*, *ABLIM1*, *PGBD5*, *PIWIL1*, *TEX26*, *TEX26-AS1*, *DYNLT4*, *BTBD19*, *DLL1*, *DSE*
**Immune/inflammatory and barrier-related pathways** *CSF1*, *TRIM14*, *LY6E*, *SBNO2*, *ABCA7*, *B3GALT4*, *CCR1*, *PTPRJ*, *MUC2*, *VMP1*, *MCRIP1*, *ADAMTS2*, *RUNX3*
**Mitochondrial, lysosomal and metabolic pathways** *NDUFS7*, *SNN*, *PLA2G15*, *APOB*, *NAA16*, *MOB3A*

**Table 5:** List of techniques that can be used to measure endophenotypes.

Endophenotype	What It Captures	Technique Families
Biochemical	Neurotransmission and molecular signalling	Magnetic resonance spectroscopy (MRS) Molecular imaging (PET/SPECT) Biofluid biomarker assays Cell-based/iPSC-derived neuronal assays
Physiological	Circuit excitability and timing	Electroencephalogram/Magnetoencephalography (EEG/MEG) Electromyography Functional Positron Emission Tomography (fPET) Functional Near-Infrared Spectroscopy (fNIRS)
Structural	Morphometry and microstructure	Structural magnetic resonance imaging (sMRI) Diffusion magnetic resonance imaging (dMRI) Quantitative magnetic resonance imaging (qMRI)
Functional	Systems-level activation and connectivity	Task-based functional Magnetic Resonance Imaging (Task-fMRI) Resting-state functional Magnetic Resonance Imaging (Resting-state fMRI) Perfusion Arterial Spin Labelling Magnetic Resonance Imaging (ASL MRI) Positron Emission Tomography with Fluorodeoxyglucose (FDG PET)
Executive	Inhibitory control and flexibility	Neuropsychological tasks Computational assays
Cognitive	Learning and memory profiles	Memory/learning batteries Associative/extinction tasks Habit/procedural tasks

**Table 6:** Sample size and population/ancestry composition of key association studies discussed in Section 2 and Section 3.

Ref.	Study	Design	Sample Size	Population/Ancestry	Notes
[14]	Strom et al., 2025	GWAS meta-analysis	53,660 cases; 2,044,417 controls	European ancestry	Identified 30 loci; used imputed GWAS datasets.
[15]	Halvorsen et al., 2025	CNV burden analysis (microarray)	2248 cases; 3608 controls	Sweden and Norway (Scandinavian/European)	Rare CNVs ≥ 30 kb; genotype array CNV calling.
[16]	Abdallah et al., 2025	De novo CNVs from WES (paediatric OCD)	183 OCD trio families; 771 control families	Multi-site; ancestry described as diverse in secondary reports	CNV calling from WES; focus on de novo CNVs.
[18]	Cappi et al., 2020	De novo damaging coding variants (trio exomes)	222 OCD trios; 855 unaffected control trios (QC subsets: 184/777)	Multi-site; ancestry not fully specified in abstract	Overlap with Tourette syndrome and autism genes.
[19]	Halvorsen et al., 2021	Whole-exome sequencing (rare coding variants)	Total 1313 cases (587 trios, 41 quartets, 644 singletons); case–control: 1263 cases vs. 11,580 controls	Diverse ancestry (multi-cohort)	Suggestive SLITRK5 signal; loss-of-function burden in vulnerable genes.
[20]	Cappi et al., 2016	WES (de novo coding variants; pilot)	20 OCD trios	Not reported in abstract (multi-site clinical cohorts)	Early WES trio study in OCD.
[21]	Lin et al., 2022	Whole-genome sequencing (de novo variants)	53 parent-offspring families (paediatric-onset OCD probands)	China (Shanghai clinical cohort; likely Han Chinese)	De novo variants implicating chromatin modification pathways.
[29]	Stewart et al., 2013	GWAS (case–control + family-based)	1465 cases; 5557 controls; 400 trios	European ancestry + Afrikaner (South Africa) + Ashkenazi Jewish (multi-ancestry cohort)	Genotyping arrays; first GWAS in OCD.
[33]	Burton et al., 2021	GWAS of paediatric OC traits (community cohort)	5018 unrelated children	Predominantly Caucasian/European ancestry (Canada; TOCS cohort)	Trait-based GWAS (symptom dimensions), not clinical OCD diagnosis.
[34]	McGrath et al., 2014	CNV analysis (cross-disorder OCD/TS)	1613 OCD cases; 1789 controls (plus 1086 TS cases)	Multi-site; ancestry not uniformly reported in abstract	Large, rare CNVs > 500 kb; cross-disorder design.
[38]	Yue et al., 2016	Epigenome-wide DNA methylation (blood; 450K)	65 cases; 96 controls	China (clinical sample; likely Han Chinese)	Illumina 450K; 8417 differentially methylated probes reported.
[39]	D’Addario et al., 2016	Candidate methylation (OXTR gene)	42 cases; 31 controls	Italy (European clinical sample)	OXTR methylation/hydroxymethylation in blood; exploratory candidate approach.
[41]	Schiele et al., 2022	EWAS (blood; EPIC)	76 cases; 76 controls	European ancestry	Illumina EPIC array; epigenome-wide differential methylation.
[42]	Campos-Martin et al., 2023	Epigenome-wide analysis (blood; EPIC)	185 cases; 199 controls	Germany (European)	Multi-site German recruitment; methylome profiles linked to OCD.
[43]	Hoffler et al., 2025	MWAS (saliva; EPICv2; preprint)	414 cases; 384 controls	Scandinavia (likely Norway; clinical cohorts)	Saliva DNA methylation; EPICv2 (Illumina) platform.
[45]	Yue et al., 2020	miRNA candidate biomarker study (plasma)	30 cases; 32 controls	China	miR-132 and miR-134 expression; case–control design.
[46]	Aydin et al., 2022	miRNA and treatment resistance (SSRI)	100 cases; 50 controls	Turkey	Assessed whether miRNA expression predicts SSRI treatment resistance.
[47]	Korkmaz et al., 2025	miRNA + monoamine markers (female-only)	22 cases; 20 controls (female)	Turkey	Female-only sample; serotonin/dopamine activity plus miRNAs.
[48]	Altunoz et al., 2025	TGF-beta signalling + miR-132 (serum)	48 cases; 48 controls	Not explicitly reported in abstract	Integrated cytokine (TGF-beta) and miRNA measures.
[49]	Aydin et al., 2025	Executive functions + miRNA (case–control)	70 cases; 35 controls	Turkey	Cognitive testing alongside miRNA measures.

## Data Availability

No new data were created or analysed in this study.

## Abbreviations

OCD	Obsessive–Compulsive Disorder
GWAS	Genome Wide Association Study
CNV	Copy Number Variation
WES	Whole Exome Sequencing
WGS	Whole Genome Sequencing
MWAS	Methylome-Wide Association Studies
EWAS	Epigenomic-Wide Association Studies
CSTC	Cortico–Striato–Thalamo–Cortical
PFC	Prefrontal cortex
EEG	Electroencephalogram
MRS	Magnetic Resonance Spectroscopy
PET	Positron Emission Tomography
MHC	Major Histocompatibility Complex
NO	Nitric Oxide
OFC	Orbitofrontal Cortex
DMP	Differentially Methylated Position
DMR	Differentially Methylated Region
